# Concurrence of cat-scratch disease and paradoxical tuberculosis-IRIS lymphadenopathy: a case report

**DOI:** 10.1186/s12879-022-07170-3

**Published:** 2022-03-03

**Authors:** Gerasimos Eleftheriotis, Elias Skopelitis

**Affiliations:** 2nd Department of Internal Medicine, General Hospital of Nikaia-Piraeus “Agios Panteleimon”, 3 Andrea Petrou Mantouvalou Street, 185 43 Athens, Greece

**Keywords:** Case report, HIV, Tuberculosis, *Bartonella*, Immune reconstitution inflammatory syndrome

## Abstract

**Background:**

Mycobacterial infections can cause significant morbidity when cellular immunity is compromised. Patients with AIDS can be affected directly from infection or through mycobacterial IRIS, especially if they are previously untreated for HIV. Herein a case of tuberculous lymphadenitis is reported, which primarily responded to antimicrobials but complicated by IRIS and cat-scratch disease at a later course.

**Case presentation:**

A 23-year-old man, intravenous drug user with untreated HIV and HCV infection presented with fever and painful cervical lymphadenopathy. *Mycobacterium tuberculosis* was isolated from PCR and culture of ultrasound-guided lymph node aspirate and a four-drug anti-TB regimen was subsequently administered, leading to complete resolution of clinical and laboratory abnormalities. Given the patient’s CD4 count (67 cells per mm^3^), antiretroviral treatment started seven weeks after TB treatment initiation. Within the first month of ART fever recurred along with cervical lymph node inflammation. Paradoxical IRIS was considered as the most probable diagnosis but workup expanded, revealing acute *Bartonella* infection. A posteriori, the patient remembered being scratched by a stray cat two weeks before his new symptoms started. Doxycycline and corticosteroid monotherapy failed to resolve symptoms, whereas a combination of doxycycline for 3 months and methylprednisolone with long-term tapering led to negative follow-up *Bartonella* antibodies and complete clinical and biochemical response, without recurrence.

**Conclusions:**

Co-infection with TB and *Bartonella* presenting with lymphadenitis is unusual. Cat-scratch disease can be a rare clinical presentation of *Bartonella* infection in patients with AIDS, but coexistence of bartonellosis and paradoxical IRIS has never been reported before. However, physicians treating people living with HIV should be aware of this potential concurrence. Early testing for *Bartonella* infection could be offered in patients with TB and HIV co-infection in case of acute deterioration or partial response to treatment, especially if they have a history of cat exposure, since clinical picture can be indistinguishable.

## Background

TB is still the leading cause of HIV-associated illness and mortality worldwide. Mycobacterial infection in treatment-naive HIV patients can cause major complications; after starting ART, IRIS can also occur. IRIS etiology includes concurrent AIDS-associated infections or other diseases like Kaposi sarcoma and usually develops within the first 3 months of ART, when blood CD4 counts start to rise. These disorders can be either subclinical, escaping from diagnosis during ART initiation and become clinically apparent afterwards, as a result of augmented immune response (unmasking IRIS) or already managed prior to ART initiation, with IRIS presenting as acute symptom worsening, while the patient is otherwise improving (paradoxical IRIS).

*Bartonella* infections are also included in AIDS-associated disorders, presented mainly as bacillary angiomatosis, peliosis hepatis, osteomyelitis and endocarditis. Albeit rare, cases of *Bartonella* infection presenting with lymphadenopathy without other organ manifestations have also been reported in HIV patients, resembling cat-scratch disease [1–3]. *Bartonella* infection in immunocompetent individuals usually has a self-limited clinical course with gradual total resolution of lymphadenopathy within 2–6 months. Management includes supportive measures, antibiotics like azithromycin that are active against atypical pathogens, and even surgery for refractory, bulky lymph node disease.

## Case presentation

A 23-year-old Caucasian man presented to the emergency department due to fever along with malaise over a 9-day period, and worsening neck pain and painful swallowing over the previous month. For these symptoms he had been examined at an outpatient clinic; amoxicillin-clavulanate was prescribed, with no clinical amelioration. The patient was of Georgian origin and had been living in Greece since last 5 years, without having traveled abroad thereafter.

Asking for his medical history, the patient admitted intravenous drug use and HIV and HCV infection diagnosis 2 months earlier without treatment, but denied special alimentary habits, recent travel at the countryside and close contact with people who had similar symptoms or animals. He was married without children; his wife had been recently tested positive for HIV, too, and already received ART.

Vital signs upon presentation were as follows: 120 heart beats per minute, axillary temperature 39.2 °C, blood pressure 100/50 mm Hg, oxygen saturation 95% and 18 breaths per minute. Physical examination revealed multiple big, painful and fixed bilateral cervical lymph nodes, as well as smaller (≤ 2 cm^2^) and mobile inguinal and axillary lymph nodes. Chest radiograph, as well as laryngoscopic and funduscopic examination from ENT and ophthalmologists respectively were normal.

**Fig. 1:**
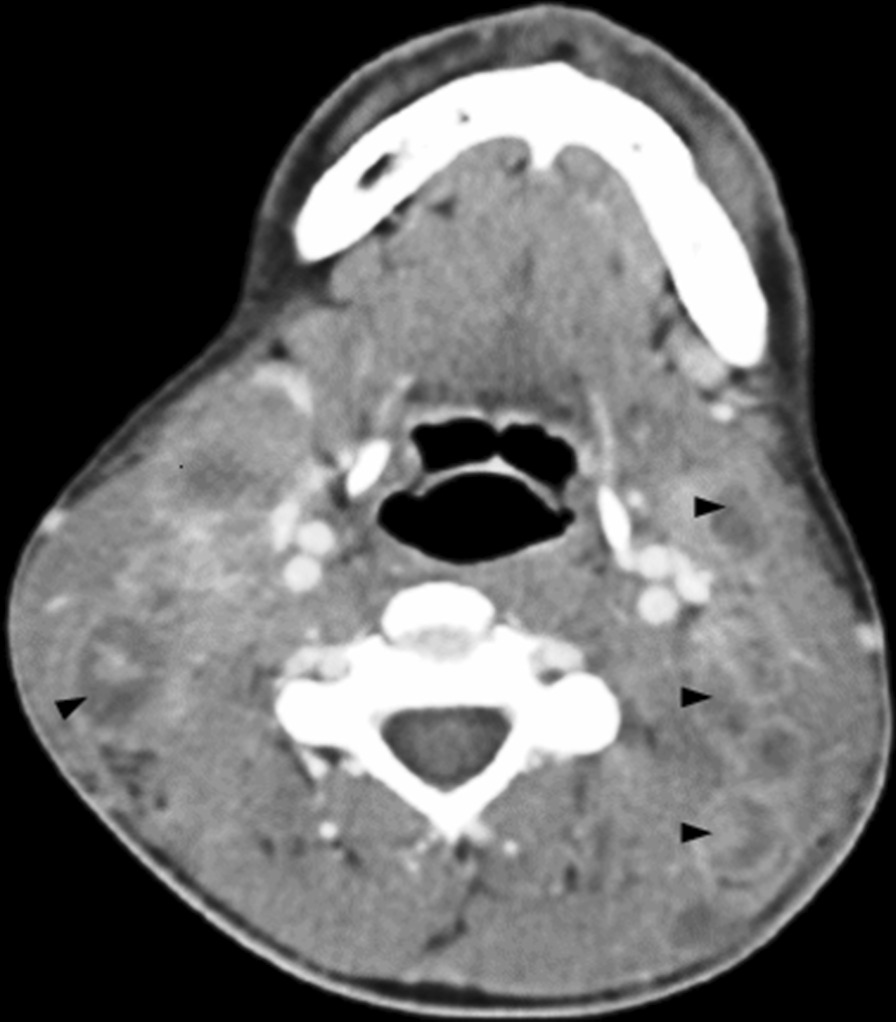
Contrast-enhanced computed tomography of the neck on first admission, revealing bilateral enlarged lymph nodes with central necrosis due to tuberculous lymphadenitis (arrowheads)

Complete blood count and biochemistry revealed normocytic, normochromic anemia and elevated LDH and CRP (Table 1). Anemia was attributed to inflammation, as low serum iron and elevated serum ferritin levels were found. Peripheral blood CD4 and CD8 counts were 67 and 773 cells per mm^3^, respectively. CT of the neck and chest was performed, showing multiple, enlarged cervical lymph nodes, some of them with signs of central necrosis (Fig. 1).

**Table 1:** Laboratory data

Variable	Reference range, adult men	On first Admission	First admission, 4^th^ hospital day	5 days after initiation of anti-TB drugs (day 12)	10 weeks after initiation of anti-TB drugs (day 77)	On second admission (day 86)	4 days after initiation of doxycycline-corticosteroid combinatio n (day 106)	1 month after initiation of doxycycline-corticosteroid combination (day 132)	Follow-up after the end of treatment
Hemoglobin (g/l)	130–170	98	92	109	122	114	116	141	133
White-cell count	4–12 (× 10^9^/l)	11.64 (84.3% neutrophils, 1.32 lymphocytes)	5.15	3.28 (72.3% neutrophils, 0.59 lymphocytes)	6.37 (82% neutrophils, 0.55 lymphocytes)	17.24 (81.9% neutrophils)	9.24	5.59	9.45
Platelet count	145–415 (× 10^9^/l)	288	230	330	203	275	621	307	383
Aspartate aminotransferase (μkat/l)	0.17–0.67	0.43	0.38	0.43	0.5	0.85	0.27	0.52	0.5
Alanine aminotransferase (μkat/l)	0.17–0.67	0.23	0.27	0.3	0.58	2.2	0.37	0.77	0.67
Alkaline phosphatase (μkat/l)	0.67–2.09	0.42		0.75	1.2	1.95	1.25	1.5	1.59
Gamma-glutamyltransferase (μkat/l)	0.25–1.42	0.5			1.69	3.14	2.64	2.45	1.17
Lactate dehydrogenase (μkat/l)	1.3–3.9	6.68	5.76		3.91				3.46
C-reactive protein (mg/l)	< 5	88.2	35.9	6.3	1	131.4	15	1.4	3.2
Erythrocyte sedimentation rate (mm/hr)	< 20		113	101	16	85	86	26	7

The patient was admitted for further evaluation and treatment; IV ampicillin-sulbactam 3 g qid and clindamycin 600 mg tid were empirically administered. Despite a significant reduction of CRP values after 4 days of antibiotic treatment (Table 1), symptoms persisted and oropharyngeal candidiasis was presented. Antibiotics were discontinued and further testing for bacteria, fungi, parasites and immune-mediated disorders was carried out (Table 2). The patient received oral fluconazole 100 mg qd for two weeks with subsequent resolution of mycosis without recurrences. Tuberculin skin testing was non-reactive.

**Table 2:** Laboratory Data

Variable	Reference range, adults	1st admission, 7th hospital day	Rest of serology and molecular studies
α_1_ globulins (g/l)	2–4	6	*Mycoplasma pneumoniae*, EBV, CMV antibodies: IgM negative, IgG positive
α_2_ globulins (g/l)	5–9	10	*Bartonella henselae*, *Coxiella burnetii*, *Chlamydia pneumoniae, Leishmania* spp, *Toxoplasma gondii,* parvovirus*,* coxsackie virus antibodies: IgM and IgG negative
β globulins (g/l)	6–11	12	
IgG (g/l)	7–16	18.9	Antinuclear antibodies, Brucella agglutination tests (Rose-Bengal and Wright), RPR, serum cryptococcal antigen: all negative
IgA (g/l)	0.7– 4	7.46	
IgM (g/l)	0.4– 2.3	1.25	
Angiotensin -converting enzyme (μkat/l)	0.15–1.1	0.87	

The ultrasound-guided fine needle lymph node aspiration performed by the Department of Radiology drained purulent fluid. Gram stain was negative, but Ziehl–Neelsen stain demonstrated acid-fast bacteria. Oral treatment with isoniazid 300 mg, rifampicin 600 mg, pyrazinamide 1500 mg, ethambutol 1500 mg and pyridoxine 50 mg once daily was initiated, leading to defervescence after 3 days and improvement of lymph node pain and laboratory parameters (Table 1). PCR and culture of the aspirate isolated a pan-susceptible *Mycobacterium tuberculosis* strain. Blood culture for mycobacteria was sterile.

In order to evaluate the extent of disease, the patient underwent abdominal CT and cerebral MRI, with no abnormal findings. He was discharged after 21 days, receiving anti-TB treatment and trimethoprim/sulfamethoxazole (800 + 160) mg thrice weekly for *Pneumocystis jirovecii* prophylaxis. Follow-up included frequent visits at the Infectious Diseases clinic. Lymph node tenderness resolved totally after approximately 1 month of treatment and lymph node size decreased. Pretreatment HIV-1 RNA viral load was 3,200,000 copies/ml, whereas molecular resistance to saquinavir, atanazavir and nevirapine was detected. Screening for the HLA-B*57:01 was negative. ART was started after seven weeks of anti-TB chemotherapy, consisting of TDF/emtricitabine 245 mg/200 mg fixed combination qd and dolutegravir 50 mg bid.

**Fig. 2:**
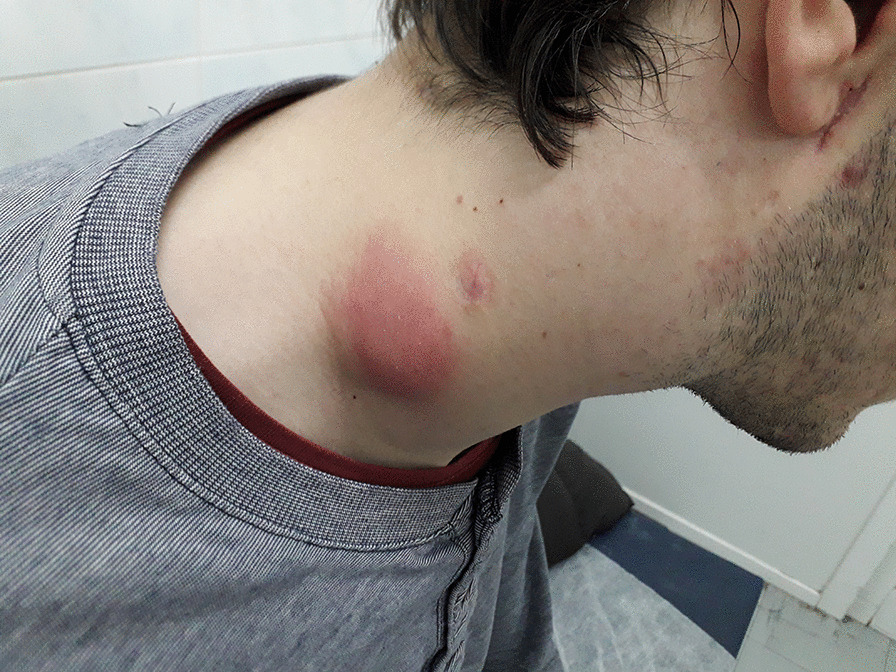
Photograph on second admission, showing cervical lymphadenopathy with redness of the overlying skin caused by tuberculosis-IRIS and acute *Bartonella* infection

Approximately 1 month after ART initiation the patient presented at the clinic due to intermittent fever and fatigue of 7-day duration and bilateral painful cervical lymphadenopathy of 4-day duration. Lymph nodes were fixed and tender and the overlying skin was reddish and warm (Fig. 2). Laboratory tests showed elevation of inflammatory markers and liver enzymes (Table 1). Chest radiograph had no abnormalities.

The patient was readmitted and cefuroxime 1.5 g tid plus doxycycline 100 mg bid were administered. A new peripheral blood flow cytometry revealed significantly higher CD4 and CD8 counts (350 and 1215 cells per mm^3^ respectively). Blood cultures were obtained and underwent prolonged incubation, whereas other tests were ordered, too; serum *Toxoplasma gondii* and *Bartonella* antibodies and blood PCR for EBV, CMV and adenovirus. Three days of antibiotic treatment did not improve patient’s symptoms; thus, they were discontinued. The patient was discharged and methylprednisolone 1.2 mg/kg daily split in two doses (equal to prednisolone 1.5 mg/kg) was prescribed along with paracetamol and etoricoxib for pain management with a working diagnosis of paradoxical TB-IRIS, while tests’ results were pending. That regimen led to moderate improvement of general condition and lower fever spikes but not to defervescence.

Blood PCR for CMV was positive; viral load was low, however, (309 copies/ml), so a CMV reactivation was excluded. The rest of the tests were negative, except of positive *Bartonella henselae* and *Bartonella quintana* IgM antibodies on a titer of 1/24 and IgG antibodies on a titer of 1/256, performed with IFA. After repeated questioning, the patient remembered a minor scratch and bite from a stray cat approximately two weeks before the new episodes of fever started. Funduscopic examination and abdominal ultrasound in order to exclude liver and spleen peliosis were performed; both were unremarkable except of mild splenomegaly, with maximum craniocaudal splenic length being 13.3 cm, compared to 11.9 cm 3 months earlier. A transesophageal cardiac ultrasound was offered as diagnostic workup for concomitant *Bartonella* endocarditis, but the patient refused. Doxycycline was reinitiated instead of corticosteroids but patient’s cervical pain and fever were not improving after 5 days.

**Fig. 3:**
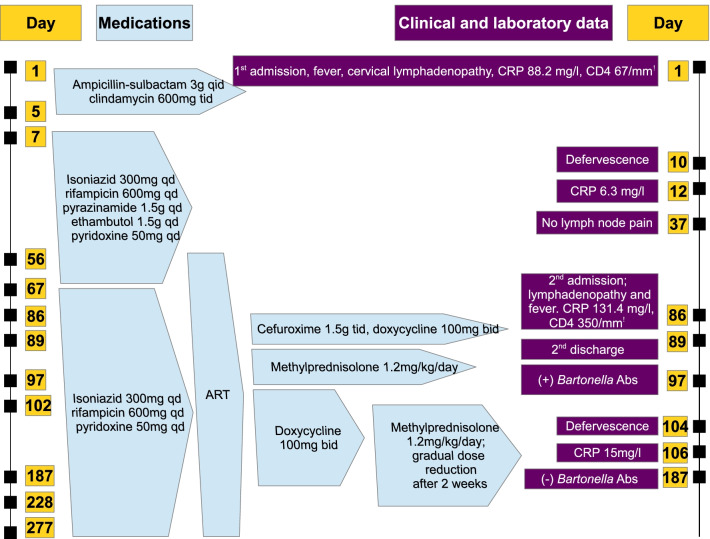
Summary of the patient’s clinical course as a timeline. CRP, C-reactive protein; ART, antiretroviral therapy; Abs, antibodies

A diagnosis of *Bartonella henselae* infection along with IRIS was consequently made; a combination of doxycycline 100 mg bid and methylprednisolone 1.2 mg/kg daily was opted, leading to resolution of symptoms and gradual normalization of laboratory parameters (Table 1). Corticosteroid dosage was reduced by half after two weeks, followed by gradual tapering within 18 weeks in total. Three months after doxycycline initiation another measurement of serum *Bartonella* antibodies was obtained; antibodies were negative, and doxycycline was subsequently discontinued. The patient also completed a 7-month isoniazid-rifampicin combination after a 2-month, four-drug anti-TB regimen. He remained asymptomatic during follow-up and laboratory parameters were within normal limits after cessation of antimicrobials against TB and *Bartonella* (Table 1). A brief summary of the patient’s clinical course, main laboratory findings and therapeutic management organized as a timeline is available in Fig. 3.

## Discussion

The case reported herein describes a patient with TB lymphadenopathy complicated by *Bartonella* lymphadenopathy and IRIS. To our knowledge, there is only one well-documented case of lymphadenopathy from co-infection with *Mycobacterium tuberculosis* and *Bartonella* in the literature, but without IRIS [4]. In that case, which was published in 1999, a 32-year-old female patient with newly diagnosed HIV infection and CD4 counts 416 cells/mm^3^ presented with painful supraclavicular lymphadenopathy. She underwent en bloc resection of the mass, and *Bartonella quintana* και *Mycobacterium tuberculosis* were isolated from tissue culture. Notably, the patient had no history of exposure to lice or cats. Anti-TB regimen and zidovudine plus lamivudine were administered postoperatively, but no antibiotic against *Bartonella*. However, she had previously received doxycycline for 10 days as an empiric treatment. One-year follow-up showed no complications or relapse of *Bartonella quintana* infection.

Bartonellosis and paradoxical IRIS coexistence has never been previously published, too; nevertheless, association with unmasking IRIS has been anecdotally found. Abino et al. reported a 35-year-old woman with AIDS (CD4 counts 23 cells/mm^3^) hospitalized for HIV encephalitis [5]. PI-based ART was administered, consisting of zidovudine, lamivudine and indinavir, and led to a substantial improvement. After hospital discharge, the patient had regular contact with a cat, which was the reason she presented fever and splenitis due to *Bartonella henselae* requiring readmission; she was managed with splenectomy and doxycycline. The case was considered as *Bartonella*-associated unmasking IRIS because CD4 counts had been increased to 109 cells/mm^3^ at the onset of symptoms and histological findings from splenic tissue (necrotizing granulomas) were compatible with immune restoration; features of bacillary peliosis in immunocompromised patients, like endothelial cell proliferation, were absent. Mejía et al., on the other hand, published the case of a 29-year-old man with a history of cat exposure and AIDS (CD4 counts 14 cells/mm^3^) that developed bacillary angiomatosis lesions 15 days after ART initiation [6]. The patient was treated with doxycycline until CD4 counts increased to > 200 cells/mm^3^, resulting in complete resolution of symptoms.

IDSA guidelines suggest initiation of ART within the first two weeks of starting anti-TB treatment in patients with CD4 counts < 50 cells/mm^3^ and within the first eight weeks to the rest except those having TB of the central nervous system, where ART should be further delayed [7]. First-line ART regimens in patients who also receive rifampicin include two NRTIs plus either efavirenz or an integrase inhibitor (dolutegravir or raltegravir) [8]. Results from an upcoming trial comparing efavirenz and dolutegravir-containing regimens in people living with HIV and TB are expected in the future [9].

Antibiotics were empirically administered initially in both admissions because bacterial agents like *Staphylococcus aureus* and *Streptococcus* spp can also cause fever and regional lymphadenopathy and rapidly evolve to sepsis if left untreated, given the patient’s immunocompromised status [10].

Concerning *Bartonella* infection diagnosis, serology is the most accessible method in most settings. Although positive serology has good specificity, negative predictive value ranges between 54 and 74%, depending on which subclass (IgM or IgG) and assay is used [11]. Seropositivity for *Bartonella* between asymptomatic people living with HIV is not negligible, too (22.3% at a publication from Spain), whereas cross-reactivity between *Bartonella* subspecies antibodies is commonly found [12]. *Bartonella henselae* was considered as the most probable causative agent in that case because of clinical picture, although *Bartonella quintana* may also cause lymphadenitis and infect cats and cat flea.

Several serologic cut-offs have been used for cat scratch disease diagnosis. IgM and/or IgG positive antibodies with a titer ≥ 1:256, IgM ≥ 1/80 or IgG ≥ 1/512 or fourfold increase in IgM and/or IgG in two consecutive samples, IgG ≥ 1: 64 with IFA [13–15]. The case reported here fulfilled all three aforementioned criteria because *Bartonella* antibodies were negative at initial presentation, thus making cat-scratch disease a definite diagnosis. On the other hand, cat-scratch disease presentation on that patient had similar clinical signs with the initial presentation of TB lymphadenitis, favoring, to our opinion, paradoxical TB-IRIS as a concurrent clinical entity in that case.

Concerning antimicrobial chemotherapy, treating cat-scratch disease with antibiotics can shorten days of illness for approximately 11 weeks and possibly prevent other organ complications [16]. A 3-month doxycycline regimen was opted with negative follow-up antibodies aiding in antibiotic cessation, extrapolating IDSA guidelines for *Bartonella* infections in HIV patients, along with methylprednisolone [7]. Corticosteroids have been also anecdotally used for severe or non-responsive to antibiotics cat-scratch disease and seem to improve both systemic symptoms and lymph node inflammation, thereby minimizing the need for surgical management in such cases [17, 18].

Glucocorticoids are also one of the mainstays of TB-IRIS management; use of other agents like anakinra and anti-TNFα have been also reported in steroid-resistant cases [19, 20]. Of note, the patient presented IRIS even if ART was started seven weeks after anti-TB drugs. Corticosteroid treatment was essential in order to achieve a positive outcome to our opinion; there is no data, however, supporting corticosteroid prophylaxis for that patient. Such evidence exists only for patients with pretreatment CD4 counts < 100 cells/mm^3^ and viral load > 100,000 copies/ml who start ART within 1 month of anti-TB drugs [21]. Future studies should assess the value of prophylactic corticosteroids in patients with CD4 counts > 100 cells/mm^3^ or when ART is commenced after the first month of anti-TB treatment.

## Conclusions

*Bartonella* infection can present as cat-scratch disease in patients with HIV infection and even coexist with tuberculosis. Paradoxical IRIS was triggered by this concurrence, which had not been previously associated with bartonellosis in the literature. Presence of risk factors for *Bartonella* infection should alert physicians treating people living with HIV for these potential complications and thus promptly perform the appropriate diagnostic workup*,* since cat-scratch disease cannot be differentiated on clinical grounds from TB lymphadenitis or TB-IRIS. Testing for *Bartonella* could also be ordered in patients with TB and HIV co-infection who are partial responders to antitubercular therapy or present paradoxical exacerbation of symptoms. Future studies will possibly assess if the role of prophylactic corticosteroids in treatment-naïve patients with AIDS receiving anti-TB treatment should be expanded.

## Data Availability

All data generated or analyzed during this study are included in this published article.

## Abbreviations

IRIS: Immune reconstitution inflammatory syndrome
AIDS: Acquired immune deficiency syndrome
HIV: Human immunodeficiency virus
HCV: Hepatitis c virus
PCR: Polymerase chain reaction
TB: Tuberculosis
ART: Antiretroviral therapy
LDH: Lactate dehydrogenase
CRP: C-reactive protein
CT: Computed tomography
IV: Intravenous
MRI: Magnetic resonance imaging
TDF: Tenofovir disoproxil fumarate
EBV: Epstein–Barr virus
CMV: Cytomegalovirus
IFA: Indirect fluorescence assay
PI: Protease inhibitor
NRTI: Nucleoside reverse transcriptase inhibitor
TNF: Tumor necrosis factor
